# Current Understanding of Breast Implant-Associated Anaplastic Large Cell Lymphoma

**DOI:** 10.7759/cureus.30516

**Published:** 2022-10-20

**Authors:** Chehak Bewtra, Pankaj Gharde

**Affiliations:** 1 Department of Surgery, Jawaharlal Nehru Medical College, Datta Meghe Institute of Medical Sciences, Wardha, IND

**Keywords:** anaplastic lymphoma kinase, cd30, anaplastic, anaplastic large cell lymphoma (alcl), breast cancer, lymphoma, breast implants

## Abstract

Every year, breast implants are inserted worldwide for reconstructive or aesthetic reasons. Breast implant-associated anaplastic large cell lymphoma (BIA-ALCL) is a rather uncommon type of T cell lymphoma that is positive for the CD30 biomarker. Despite being far more common than other primary breast lymphomas, BIA-ALCL has a very low incidence. Textured types of implants have been linked to almost all cases. The majority of patients have a favorable prognosis after the removal of implants and capsules. Nevertheless, the chance of a fatal outcome is higher with capsular invasion and tumor bulk. Although the exact cause of BIA-ALCL is unknown, it has been suggested that persistent infections or toxins related to the implants may play a role. Therefore, physicians must be aware of breast implants' rare but potentially significant side effects. Before surgery, patients with verified instances should be directed to a breast medical oncologist or lymphoma specialist for oncologic assessment. Total en-bloc capsulectomy, a surgical procedure that removes the implant and the surrounding capsule, is usually adequate. In other cases, a late diagnosis necessitates more invasive surgery and systemic therapies, which, while typically effective, have been linked to poor outcomes and even fatalities. Since it is a recently described entity and the "denominator" (i.e., the total number of breast implant procedures) is unknown, it is challenging to determine the risk of development. In this review, we hope to emphasize the elements of etiology, demography, clinical features, and current treatment approaches for BIA-ALCL. In doing so, we hope to increase the medical professional's knowledge of the recognition and treatment of BIA-ALCL.

## Introduction and background

Breast implants have been a standard procedure to augment and reconstruct breasts all around the world in recent years [1]. Annually, breast implants are given to more than 1.5 million women around the world [2]. An uncommon subtype of T cell non-Hodgkin lymphoma, called breast implant-associated anaplastic large cell lymphoma (BIA-ALCL), typically develops in the fluid and the encircling capsule [3]. Keech and Creech, in 1997, mentioned the patient with a McGhan textured saline implant who had the first case of BIA-ALCL and displayed a 2-cm tumor that significantly affected the capsule. Following a complete capsulectomy and modification of the implant, chemotherapy and radiotherapy were administered. According to reports, the patient was tumor-free two years later [4]. Less than 1,000 cases and 36 fatalities have been reported to date, and the average patient presents seven to 10 years after the initial insertion of the breast implants [3,5]. The American Food and Drug Administration (FDA) revealed a potential connection between breast implants and the emergence of BIA-ALCL in January 2011 [6,7]. It was introduced as a provisional entity to the WHO Classification of Tumors of Hematopoietic and Lymphoid Tissues [8-10]. The FDA, in association with the American Society of Plastic Surgeons and the Plastic Surgery Foundation, created the Patient Registry and Outcomes for Breast Implants and Anaplastic Large Cell Lymphoma Etiology and Epidemiology (PROFILE) registry, which finds more information to further define it [11,12]. The registry's objective is to gather reliable patient data, such as (but not exclusively) pathophysiology, factors at risk, and demographics, to encourage and support future research on the understanding of the condition [13]. By June 2022, the (PROFILE) registry has received reports of 389 suspected or confirmed BIA-ALCL cases in the United States [5]. BIA-ALCL is frequently found many years after the breast prosthesis was placed, with the mean period following implant surgery being about 10 years, which is suggestive of its indolent character [14,15]. Despite advances in science, BIA-ALCL has a favorable prognosis and a relatively low mortality rate compared to other cancers [16]. Anaplastic large cell lymphoma associated with breast implants is still uncommon, and the risk factors are still unclear. To increase our understanding of this rare disease, ongoing data gathering and collaborative care are crucial [17]. This review summarizes the most significant BIA-ALCL-related findings and identifies the main areas that we think require additional study.

## Review

Epidemiology

Numerous research conducted over the last 20 years has demonstrated that BIA-ALCL is a distinct type of invasive lymphoid tumor and that the rate of occurrence differs significantly around the globe [1]. The FDA website update from August 20, 2020, notes that while it is still challenging to pinpoint the precise number of BIA-ALCL cases, a thorough investigation has revealed that there have been 733 distinct confirmed cases worldwide, including 36 reported deaths [5,18]. The lifetime chance of developing BIA-ALCL is 1:30,000 in women who had breast implants that were textured, according to research [19]. Brody et al. analyzed the whole of recent BIA-ALCL research, including 173 cases of the condition. The demographics, accompanying skin lesions, and tissue culture data showed a probable unusual genetic susceptibility. The common characteristics appear to be the texturing of the silicone breast implant surface, suggesting a site and material-specific chronic inflammatory etiology with biofilm organisms as potential participators. Furthermore, there were no reports of the illness before the invention of surface-textured implants [1,4,15].

It has been challenging to estimate the incidence and frequency of BIA-ALCL due to its rarity and patchy reporting. An odds ratio of 18.2 indicates that individuals with implants were 18 times more likely to develop the disease than patients without breast implants in a Dutch-based study conducted in 2008 that revealed a link between implants and the disease [20,21]. It has received significant public and media focus despite its rarity. In December 2018, France's National Agency for the Safety of Medicines and Health Products (ANSM) and Health Canada initiated the review and outlawed the sale of macro-textured implants which were linked to BIA-ALCL, and a voluntary recall was ordered across 33 nations [3]. The prohibition is being implemented by the ANSM as a "precautionary step", even though it has not established a causal connection between BIA-ALCL and textured breast implants. Furthermore, all implant manufacturers doing business in France were urged by ANSM to provide comprehensive safety information for textured implants within the next year, failing which their products will not be sold in France [2]. Other manufacturing companies' textured implants have also been linked to cases, and textured implants with a large surface area were linked to an increased chance of BIA-ALCL. Due to this, the FDA decided to utilize standardized informed consent in 2020 and to issue a disclaimer with a black box for breast implant packages and also stated that despite the new warning, it does not advise women to remove their breast implants. However, the organization advises women to keep an eye on their implants and to seek medical attention if they observe any unusual changes [3,22].

Etiology and pathogenesis

Although the pathophysiology of BIA-ALCL is not fully known, current research is consistent with the notion which states that the development of BIA-ALCL is probably a complicated system with many etiology [13]. All documented cases with textured implants [13,23] were originally launched during the 1970s with a coating made of polyurethane which increased the likelihood of contraction of the capsule and BIA-ALCL due to bacterial attachment. Later in the 1980s, silicone-filled textured implants were introduced in an effort to lessen the likelihood of contraction of the capsule [24,25]. Clinically, implant texturing may lower the risk of capsular contracture after augmentation and increase implant stability on the chest wall. Emerging data, however, shows that the majority of BIA-ALCL cases were recorded in patients who had textured implants, suggesting that implant texturization may be a risk factor for the development of BIA-ALCL [1]. According to Loch-Wilkinson et al., Siltex textures (Johnson & Johnson, New Brunswick, New Jersey) have a lower surface area than polyurethane (Silimed, Rio de Janeiro, Brazil) and Biocell textures (Allergan, Dublin, Ireland). In Australia and New Zealand, Polyurethane (Silimed) textured implants had a 10.84 times greater risk of BIA-ALCL compared to Biocell textured implants, which had a 14.11 times higher risk [22].

Uncertainty exists regarding the pathophysiology of BIA-ALCL. According to the majority of data, breast implants cause chronic immunological stimulation. Here, we examine the immunity-mediated features of BIA-ALCL with its possible initiating factors.

Immunological Factors

**Figure 1:**
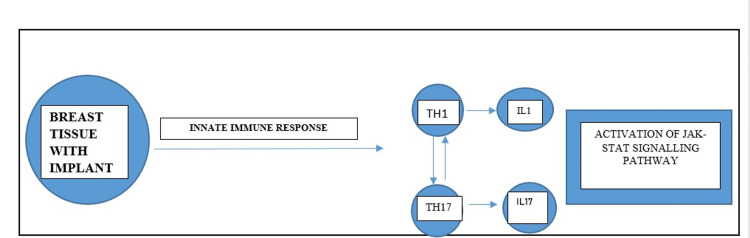
Flowchart showing possible immunological hypothesis of BIA-ALCL BIA-ALCL - breast implant-associated anaplastic large cell lymphoma The figure is created by author.

It is said that innate and adaptive immunological systems appear to work together for the creation of BIA-ALCL. In those people who have a genetic predisposition to the disease, the shell particle of the textured implants causes chronic T cell and phagocytic cellular aggregation, which results in chronic inflammation and immunological dysregulation. This collection of T cells might be in close proximity to the silicone substance, which may eventually cause them to become sensitive to particles composed of silicones or their subsequent proteins, triggering a response to the antigen and its presentation in T cells [26]. Monocytes and T lymphocytes are mainly infiltrated close to the surface of biomaterials, where they release IL-4, IL-13, and other substances as a result [6,27]. Additionally, the immunological response involves T-helper cell activation of Th1 and Th17, which helps in drawing in macrophages and attracting neutrophils to extracellular infections, respectively. A research study of whole exome sequencing performed on DNA extracted from cytology fluid and germline DNA of two patients with effusion-limited BIA-ALCL revealed mutations in JAK1 and STAT3 as well as a germline JAK3 mutation, the latter of which suggested a potential genetic susceptibility for the development of this lymphoma [28,29]. Chronic inflammation brought on by the ongoing processing of antigens may result in immunological dysregulation, cell proliferation, formation of foreign body giant cells (FBGCs) with a higher chance of genetic mutation, and ultimately cancer (Figure 1) [26].

Possible Association With Sub-clinical Bacterial Infection

Some cancer instances may be caused by infections, according to some theories. However, any direct link between bacterial infection and the disease hasn't been established. BIA-ALCL being brought on by infections is thus a possibility [6]. Recent research indicates that antigenic stimulation may be responsible for prolonging an extended period of inflammation caused by several bacteria, including *Staphylococcus* and *Ralstonia pickettii* [19,30,31]. Gram-positive bacteria (*Staphylococcus* spp.) predominated in the analysis of contracted implant capsule specimens conducted by Collett et al., while Gram-negative bacteria predominated in the analysis of BIA-ALCL (principally *Ralstonia* *pickettii* and *Pseudomonas *spp.). He came to the conclusion that Gram-positive bacteria can encourage capsular contractility, whereas Gram-negative bacteria can trigger lymphocyte simulation and, finally, transformation [15]. Even while some evidence suggests that microorganisms play a part in the etiology of BIA-ALCL, this theory has not received widespread recognition and shouldn't be presented in isolation from other aspects, such as potential genetic impacts. Viruses, one of the recently hypothesized triggers not included here, may additionally aid in the emergence of BIA-ALCL and demand more investigation [32]. The medical community will eventually be able to increase awareness and expertise with the intent of optimizing the care of the patients thanks to cooperative research on this rare disease [16].

Plausible Role of Friction of Implants

Data on various prosthetic materials used in orthopedics led to the assumption that the force of friction exerted using implants on tissue may play a function [19]. Hallab and associates, in 2019, concentrated on the connection between the sustained period of inflammation in BIA-ALCL and orthopedic implants. Orthopedic implant wear generates debris, which stimulates macrophages, causes them to phagocytose, activates inflammasomes, and releases cytokines from the interleukin family. In animal models, particles made up of silicone can cause a comparable inflammatory pattern, but this causes lesser reactions than orthopedic implants made of metal, and there is no proof that silicone orthopedic implants contain an analog of BIA-ALCL. Because of this, other mechanisms need to be researched [33].

Can the Three Factors of Texture, Friction, and Biofilm Be Connected to One Another?

Efanov et al. investigated double-capsule specimens in 2017 and proposed a mechanical representation of their origin. The breast implant is constantly under involuntary stress; in textured implants, a separation from nearby tissue might occur, while the macrotexture dictates disorganization and fractures in the collagen matrix of the double capsules. As a result, a fresh delaminated capsule develops on the lateral aspect of the breast. Implants that have macro-texturing on one side and a smooth surface on the other may reduce the mechanical shear forces that led to these results. However, double capsule and late seroma associated with an anaplastic large cell lymphoma appears to be very rare [34]. 

Clinical features

The primary and most prevalent pathogenic manifestation of BIA-ALCL is in situ development of seroma. These appearances may frequently be mistaken for harmless seromas. Other form includes intrusive disease, which can involve a perceivable breast mass or tumor or lymphadenopathy without a mass. Occasionally, patients manifest with skin eruption, discomfort, contracture of the capsule, or localized involvement of lymph nodes [17]. Because lymph node involvement indicates either progressive or chronic disorder, it has a significant impact on the prognosis of BIA-ALCL. Patients without lymph node involvement have a survival rate of 97.9 percent, but cases with lymph node involvement have a much lower survival rate of only 75% [6,35]. The depth of invasion, or the extent of the disease, seems to be the strongest prognostic factor. In contrast to patients with a mass and extracapsular extension, who have a worse prognosis, a lower chance of achieving complete remission, and a higher risk for recurrence and death, patients with BIA-ALCL in effusion or confined to the fibrous capsule appear to have long-term survival with appropriate treatment [8,36]. Immunophenotyping, Epstein-Barr virus (EBV) negativity, and T cell receptor (TCR) clonal modifications are used to distinguish BIA-ALCL from other T cell lymphomas [37].

 Workup and diagnosis

A detailed guideline covering the evaluation of patients with a recent suspicion or confirmation of BIA-ALCL was compiled. The most frequent modality used to make the diagnosis is ultrasound-guided fine-needle aspiration because a major number of cases initially appear with a seroma encircling the implant [3,11]. Using ultrasound, the breasts, chest wall, and local lymph nodes should be examined in late-onset seromas (more than one year) [38]. It is significant to note that ultrasound is the recommended diagnostic tool rather than mammography because of ultrasound's superior sensitivity for detecting BIA-ALCL-associated effusion and mass [39,40]. Ultrasound-guided imaging and magnetic resonance imaging were discovered to be the most successful techniques for locating the tumor and clearly identifying them, as said by Adrada et al. when comparing and verifying the sensitivity and specificity of several imaging techniques for mass related to BIA-ALCL. The researchers found that the range values of sensitive/specific indicators for finding an effusion by magnetic resonance imaging and ultrasonography were 84%/75% and 82%/33%, respectively [13,41]. Mammography, however, can be used to check for any potential breast masses as well as diagnosis, such as breast cancer, even though it is comparatively less sensitive and specific for BIA-ALCL [42]. Computed tomography or magnetic resonance imaging scans may help with the detection of soft tissue when ultrasonography is inconclusive [38]. Magnetic resonance imaging could be useful in the planning of surgery if a mass is present. Excisional biopsies of questionable lymph nodes and positron emission tomography (PET)/CT staging to check for any metastatic involvement may be included in further workup [23,39,43].

Diagnosis and Histopathology

Aspiration of seroma fluid under ultrasound guidance using cytological methods, flow cytometry, and interpretation of the results, especially for the expression of CD30 cell surface protein, facilitates the diagnosis [44]. Malignant cells consistently express CD30 and lack ALK. CD4 and CD43 immunohistochemistry (IHC) staining is positive in more than 80% of cases. IHC staining reveals positive results for CD3, CD45, and CD2 in 30% or more of patients. In some situations, CD15 and PAX-5 results may be positive, raising the possibility of a conventional Hodgkin's disease, particularly when infiltrative illness is present [17]. Large pleomorphic cells with lots of eosinophil granules embedded in the cytoplasm along with horse-shoe shaped nuclei (considered as hallmark cells) with noticeable nucleoli may be seen as the morphological and histological feature of malignant cells of BIA-ALCL [21,26,36].

The presence of a small amount of periprosthetic fluid may be typical and frequent surrounding breast implants. Despite the fact that more liquid results in a precise diagnosis, 10 to 50 mL should be used at least to adequate material to allow for the creation of the smears [11]. Centrifugation is used to separate cells from fluid samples and create cytospins, which are then colored for better examination [42]. To allow Hematoxylin and Eosin stain and investigation of formaldehyde and wax-enclosed portions or cuts, preparation of a cell block is preferred. This is due to the possibility that a large number of the antibodies required for the confirmation of BIA-ALCL could possibly be not verified on the slide, which is left to air-dry under a cytocentrifuge [11,45]. Since breast implant-associated anaplastic large-cell lymphoma is an unusual disease entity that few institutions are familiar with, consulting a specialist in hematopathology is crucial. It is best to send suspicious pathology samples to an experienced pathologist for review. Prior to the review, the pathologist should be informed that there is a possibility that the patient has BIA-ALCL since specific cell markers that might otherwise not be requested may be useful for identification. It is also advised that if the primary examination is inconclusive, a second opinion from a hematopathologist be sought after [17]. Using BIOMED-2 primers, T cell receptor (TCR) clonal modification is the cornerstone of a molecule-based test in the assessment of potential BIA-ALCL [46]. In order to assess the degree of invasion through the capsule and into neighboring tissues, sections and sampling should be collected from mass lesions. If there are no detectable mass lesions, but there is a strong presumption of BIA-ALCL, the whole capsule needs to be sent in order to further examine for any potential subtle involvement of BIA-ALCL [13,47].

Staging

The modified version of the Ann Arbor staging system, including BIA-ALCL, has historically been employed to indicate the severity of disease in lymphoma [17]. The amendment to the Ann Arbor staging system has stage IE for illness restricted to the capsule and stage IIE for restriction to the capsule and regional lymphatics of the same side [13,48]. Most BIA-ALCL patients, or 83 to 96 percent, are classed as having stage IE disease using this modified staging approach, meaning that their disease is in an early stage [49]. The guidelines proposed by the MD Anderson Cancer Center and supported by the National Comprehensive Cancer Network (NCCN) have officially endorsed the new staging approach utilizing the classification of tumor, lymph node, and metastasis (TNM) as shown in Table 1 [3,17,44].

**Table 1:** Proposed TNM staging classification of BIA-ALCL T - tumor extent (penetration of capsule), N - lymph node, M - metastasis [3], TNM - tumor, lymph node, and metastasis, BIA-ALCL - breast implant-associated anaplastic large cell lymphoma

TNM or stage designation	Description
T1	Limited to effusion or involvement of luminal surface of the capsule
T2	Early infiltration of capsule
T3	Cell aggregates or sheets penetrating the capsule
T4	Infiltration of lymphoma beyond the capsule
N0	No involvement
N1	One regional lymph node involved
N2	More than one regional lymph node involved
M0	No distant sites involved
M1	Spread of disease to distant sites
Stages	
Stage IA	T1N0M0
Stage IB	T2N0M0
Stage IC	T3N0M0
Stage IIA	T4N0M0
Stage IIB	T1-3N1M0
Stage III	T4N1-2M0
Stage IV	T1-4N0-2M1

According to this more recent classification, patients with stage I BIA-ALCL may only have effusion-limited disease, early penetration of the capsule, or a collective mass that is capsule-limited. IA, IB, and IC patients who have stage I illness do not have metastatic disease or nodal infiltration. Stage II illness (IIA or IIB) patients may additionally have tumors that localize invasion external to the capsule or are affected by a single nodal involvement. Stage III illness is characterized by the involvement of localized invasion of lymph nodes and local tumor external to the capsule. 

Treatment

Before undergoing any surgical procedure, the patient who has been diagnosed with BIA-ALCL should be referred to a breast medical oncologist or lymphoma specialist for a thorough oncologic inspection [13]. There isn't yet a standard of care for BIA-ALCL treatment. Consideration of the advantages and disadvantages of each strategy is necessary on a case-by-case basis, with collaborative decision-making, taking into account the level of concern, the differential diagnosis, and the morbidity associated with treatments such as total en-bloc capsulectomy. This may result in a pneumothorax in up to 4% of instances, the potential for chronic pain, and serious cosmetic consequences [42].

More than 85% of those who have BIA-ALCL have stage I disease, which usually presents within the seroma, capsule, or both [3,50]. A whole en bloc surgical removal of the capsule and implant under surgical care in these patients is linked to a five-year disease-free interval and no further therapy [3]. The contralateral implant should also be taken into account during removal [17]. Additionally, due to the possibility of bilateral illness, it is advised that the contralateral implant be removed. Otherwise, a sentinel lymph node biopsy cannot rule out metastases since the breast implant capsule can drain into numerous local lymph nodes. When a clinical examination or imaging results point to the involvement of lymph nodes, the dissection of the axillary lymph nodes should be taken into account. To avoid a local recurrence, every effort should be taken to complete the surgical excision [6]. The mass should be fully removed, and the surgical margins should be examined for signs of cancer [13]. For limited-stage illness, postoperative chemotherapy, radiotherapy, or both are not deemed required [13]. The ideal course of treatment is still unknown for those who have advanced disease (stage II or higher upon presentation) [3]. An expert team of surgeons, oncologists, and radiation therapists should make the choice to include adjuvant therapy in a patient's treatment strategy. Radiation therapy or systemic chemotherapy are examples of adjuvant therapies [17]. The adjuvant setting has been documented for brentuximab vedotin as well [3,13]. As the first line of standardized therapy for systemic ALCL, the majority of cases with severe illness have been given the traditional cyclophosphamide, doxorubicin, vincristine, and prednisone (CHOP) regimen [13]. A multidisciplinary, tailored strategy should be used in every instance because our understanding of this disease is insufficient. For the first two years, patients should receive clinical follow-up every three to six months and imaging every six months [23,39].

## Conclusions

BIA-ALCL is still a rare entity that affects people who have textured breast implants. Its scarcity creates more difficulty in terms of its diagnosis. The etiology of the disease and a clear understanding of its underlying causes and still unknown and need more study. Reconstructive surgery is commonly performed on individuals who have had major life-altering events, such as the diagnosis and treatment of breast cancer, and thus may be influenced by significant emotional links. For the purpose of determining potential risks and preventing future occurrences of this disease, more research is essential. Patients should get education and have access to reliable resources so they can be well informed before providing their consent for surgery in order to keep our commitment to patient safety. All positive cases and incidents ought to be notified to the authorities and reported.

## References

[1] Wang Y, Zhang Q, Tan Y (2021). Current progress in breast implant-associated anaplastic large cell lymphoma. Front Oncol.

[2] Clemens MW, Nava MB, Rocco N, Miranda RN (2017). Understanding rare adverse sequelae of breast implants: anaplastic large-cell lymphoma, late seromas, and double capsules. Gland Surg.

[3] Mehta-Shah N, Ghione P (2022). An updated approach and understanding of breast implant-associated anaplastic large cell lymphoma. J Natl Compr Canc Netw.

[4] Brody GS, Deapen D, Taylor CR (2015). Anaplastic large cell lymphoma occurring in women with breast implants: analysis of 173 cases. Plast Reconstr Surg.

[5] (2022). BIA-ALCL physician resources. https://www.plasticsurgery.org/for-medical-professionals/health-policy/bia-alcl-physician-resources.

[6] Zhang XR, Chien PN, Nam SY, Heo CY (2022). Anaplastic large cell lymphoma: molecular pathogenesis and treatment. Cancers (Basel).

[7] Groth AK, Graf R (2020). Correction to: breast implant-associated anaplastic large cell lymphoma (BIA-ALCL) and the textured breast implant crisis. Aesthetic Plast Surg.

[8] Julien LA, Michel RP, Auger M (2020). Breast implant-associated anaplastic large cell lymphoma and effusions: a review with emphasis on the role of cytopathology. Cancer Cytopathol.

[9] Keech JA Jr, Creech BJ (1997). Anaplastic T-cell lymphoma in proximity to a saline-filled breast implant. Plast Reconstr Surg.

[10] Feldman AL, Harris NL, Stein H, Swerdlow SH, Campo E, Harris NL (2017). Feldman: WHO classification of tumours of haematopoietic... - Google Scholar. Accessed: July 23, 2022. WHO classification of tumours of haematopoietic.

[11] Jaffe ES, Ashar BS, Clemens MW (2020). Best practices guideline for the pathologic diagnosis of breast implant-associated anaplastic large-cell lymphoma. J Clin Oncol.

[12] McCarthy CM, Loyo-Berríos N, Qureshi AA (2019). Patient registry and outcomes for breast implants and anaplastic large cell lymphoma etiology and epidemiology (PROFILE): initial report of findings, 2012-2018. Plast Reconstr Surg.

[13] St Cyr TL, Pockaj BA, Northfelt DW, Craig FE, Clemens MW, Mahabir RC (2020). Breast implant-associated anaplastic large-cell lymphoma: current understanding and recommendations for management. Plast Surg (Oakv).

[14] Ionescu P, Vibert F, Amé S, Mathelin C (2021). New data on the epidemiology of breast implant-associated anaplastic large cell lymphoma. Eur J Breast Health.

[15] Collett DJ, Rakhorst H, Lennox P, Magnusson M, Cooter R, Deva AK (2019). Current risk estimate of breast implant-associated anaplastic large cell lymphoma in textured breast implants. Plast Reconstr Surg.

[16] Deva AK, Turner SD, Kadin ME (2020). Etiology of breast implant-associated anaplastic large cell lymphoma (BIA-ALCL): current directions in research. Cancers (Basel).

[17] DePaola NE, Coggins H (2019). Breast implant-associated anaplastic large cell lymphoma: what we know. J Adv Pract Oncol.

[18] Myckatyn TM, Mehta-Shah N, Duncavage E (2020). Breast implant-associated anaplastic large cell lymphoma: real, rare, but avoidable. JAMA Surg.

[19] Cuomo R (2021). The state of the art about etiopathogenetic models on breast implant associated-anaplastic large cell lymphoma (BIA-ALCL): a narrative review. J Clin Med.

[20] Doren EL, Miranda RN, Selber JC (2017). U.S. epidemiology of breast implant-associated anaplastic large cell lymphoma. Plast Reconstr Surg.

[21] de Jong D, Vasmel WL, de Boer JP, Verhave G, Barbé E, Casparie MK, van Leeuwen FE (2008). Anaplastic large-cell lymphoma in women with breast implants. JAMA.

[22] Loch-Wilkinson A, Beath KJ, Knight RJ (2017). Breast implant-associated anaplastic large cell lymphoma in Australia and New Zealand: high-surface-area textured implants are associated with increased risk. Plast Reconstr Surg.

[23] Leberfinger AN, Behar BJ, Williams NC, Rakszawski KL, Potochny JD, Mackay DR, Ravnic DJ (2017). Breast implant-associated anaplastic large cell lymphoma: a systematic review. JAMA Surg.

[24] Webb LH, Aime VL, Do A, Mossman K, Mahabir RC (2017). Textured breast implants: A closer look at the surface debris under the microscope. Plast Surg (Oakv).

[25] Lazzeri D, Agostini T, Bocci G (2011). ALK-1-negative anaplastic large cell lymphoma associated with breast implants: a new clinical entity. Clin Breast Cancer.

[26] Alotaibi S, Hamadani M, Al-Mansour M, Aljurf M (2021). Breast implant-associated anaplastic large cell lymphoma. Clin Lymphoma Myeloma Leuk.

[27] Kobayashi SD, Voyich JM, Whitney AR, DeLeo FR (2005). Spontaneous neutrophil apoptosis and regulation of cell survival by granulocyte macrophage-colony stimulating factor. J Leukoc Biol.

[28] Turner SD, Inghirami G, Miranda RN, Kadin ME (2020). Cell of origin and immunologic events in the pathogenesis of breast implant-associated anaplastic large-cell lymphoma. Am J Pathol.

[29] Blombery P, Thompson ER, Jones K (2016). Whole exome sequencing reveals activating JAK1 and STAT3 mutations in breast implant-associated anaplastic large cell lymphoma anaplastic large cell lymphoma. Haematologica.

[30] DeCoster RC, Clemens MW, Di Napoli A (2021). Cellular and molecular mechanisms of breast implant-associated anaplastic large cell lymphoma. Plast Reconstr Surg.

[31] Adams WP Jr (2016). Discussion: bacterial biofilm infection detected in breast implant-associated anaplastic large-cell lymphoma. Plast Reconstr Surg.

[32] Kang ST, Wang HC, Yang YT, Kou GH, Lo CF (2013). The DNA virus white spot syndrome virus uses an internal ribosome entry site for translation of the highly expressed nonstructural protein ICP35. J Virol.

[33] Hallab NJ, Samelko L, Hammond D (2019). The inflammatory effects of breast implant particulate shedding: comparison with orthopedic implants. Aesthet Surg J.

[34] Efanov JI, Giot JP, Fernandez J, Danino MA (2017). Breast-implant texturing associated with delamination of capsular layers: a histological analysis of the double capsule phenomenon. Ann Chir Plast Esthet.

[35] Ferrufino-Schmidt MC, Medeiros LJ, Liu H (2018). Clinicopathologic features and prognostic impact of lymph node involvement in patients with breast implant-associated anaplastic large cell lymphoma. Am J Surg Pathol.

[36] Bautista-Quach MA, Nademanee A, Weisenburger DD, Chen W, Kim YS (2013). Implant-associated primary anaplastic large-cell lymphoma with simultaneous involvement of bilateral breast capsules. Clin Breast Cancer.

[37] Swerdlow SH, Campo E, Pileri SA (2016). The 2016 revision of the World Health Organization classification of lymphoid neoplasms. Blood.

[38] Fitzal F, Turner SD, Kenner L (2019). Is breast implant-associated anaplastic large cell lymphoma a hazard of breast implant surgery?. Open Biol.

[39] Spaedy A, O'Toole D, Aylward CA (2020). Breast implant-associated anaplastic large cell lymphoma: what the primary care physician needs to know. Mo Med.

[40] Clemens MW, Miranda RN (2015). Coming of age: breast implant-associated anaplastic large cell lymphoma after 18 years of investigation. Clin Plast Surg.

[41] Adrada BE, Miranda RN, Rauch GM (2014). Breast implant-associated anaplastic large cell lymphoma: sensitivity, specificity, and findings of imaging studies in 44 patients. Breast Cancer Res Treat.

[42] Turton P, El-Sharkawi D, Lyburn I (2021). UK guidelines on the diagnosis and treatment of breast implant-associated anaplastic large cell lymphoma (BIA-ALCL) on behalf of the Medicines and Healthcare products Regulatory Agency (MHRA) Plastic, Reconstructive and Aesthetic Surgery Expert Advisory Group (PRASEAG). J Plast Reconstr Aesthet Surg.

[43] O'Neill AC, Zhong T, Hofer SO (2017). Implications of breast implant-associated anaplastic large cell lymphoma (BIA-ALCL) for breast cancer reconstruction: an update for surgical oncologists. Ann Surg Oncol.

[44] Clemens MW, Horwitz SM (2017). NCCN consensus guidelines for the diagnosis and management of breast implant-associated anaplastic large cell lymphoma. Aesthet Surg J.

[45] Barbé E, de Boer M, de Jong D (2019). A practical cytological approach to the diagnosis of breast-implant associated anaplastic large cell lymphoma. Cytopathology.

[46] van Dongen JJ, Langerak AW, Brüggemann M (2003). Design and standardization of PCR primers and protocols for detection of clonal immunoglobulin and T-cell receptor gene recombinations in suspect lymphoproliferations: report of the BIOMED-2 Concerted Action BMH4-CT98-3936. Leukemia.

[47] Talagas M, Uguen A, Charles-Petillon F (2014). Breast implant-associated anaplastic large-cell lymphoma can be a diagnostic challenge for pathologists. Acta Cytol.

[48] Cheson BD, Fisher RI, Barrington SF, Cavalli F, Schwartz LH, Zucca E, Lister TA (2014). Recommendations for initial evaluation, staging, and response assessment of Hodgkin and non-Hodgkin lymphoma: the Lugano classification. J Clin Oncol.

[49] Clemens MW, Medeiros LJ, Butler CE (2016). Complete surgical excision is essential for the management of patients with breast implant-associated anaplastic large-cell lymphoma. J Clin Oncol.

[50] Miranda RN, Aladily TN, Prince HM (2014). Breast implant-associated anaplastic large-cell lymphoma: long-term follow-up of 60 patients. J Clin Oncol.

